# Brockman, M.A., *et al.*, Human Leukocyte Antigen (HLA) Class I Down-Regulation by Human Immunodeficiency Virus Type 1 Negative Factor (HIV-1 Nef): What Might We Learn From Natural Sequence Variants? *Viruses* 2012, *4*, 1711-1730

**DOI:** 10.3390/v4102014

**Published:** 2012-10-05

**Authors:** Philip Mwimanzi, Tristan J. Markle, Takamasa Ueno, Mark A. Brockman

**Affiliations:** 1 Department of Molecular Biology and Biochemistry, Simon Fraser University, 8888 University Drive, Burnaby, British Columbia V5A 1S6, Canada; Email: philip_mwimanzi@sfu.ca (P.M.); tmarkle@sfu.ca (T.J.M.); 2 Center for AIDS Research, Kumamoto University, 2-2-1 Honjo, Chuo-ku, Kumamoto 860-0811, Japan; Email: uenotaka@kumamoto-u.ac.jp; 3 Faculty of Health Sciences, Simon Fraser University, 8888 University Drive, Burnaby, British Columbia V5A 1S6, Canada

**Figure 1:**
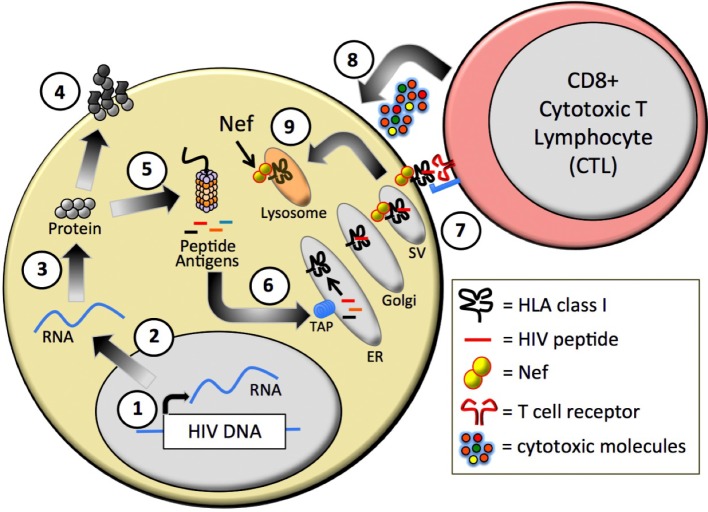
Presentation of viral peptide antigens by Human Leukocyte Antigen (HLA) class I. Human immunodeficiency virus type 1 (HIV-1) proviral gene expression, including RNA transcription (**a**) and protein translation (**b**); generates functional viral proteins (**c**) as well as truncated or mis-folded proteins that are degraded by the cellular proteasome complex to form short antigenic peptides (**d**); These peptides are transported from the cytoplasm into the endoplasmic reticulum (ER) (**e**) where they can be loaded onto HLA-I molecules.Peptide/HLA complexes traffic from the ER through the Golgi and secretory vesicle (SV) network to the plasma cell membrane, where the peptide antigens are presented to circulating cytotoxic T lymphocytes (CTL) (**f**); The viral Nef protein shuttles HLA molecules located at the cell surface or within the *trans*-Golgi network into lysosomal compartments (**g**); where they are degraded.In the absence of Nef-mediated HLA down-regulation, antigen-specific CTL respond to stimulation by releasing cytotoxic molecules, including perforin and granzymes, resulting in elimination of the virus-infected cell (h).

In the original manuscript, the text in figure 1 is illegible. Furthermore, there is an unnecessary carriage return (page 1716, ~line 18) "crystallographic ... methods".

“…The N-terminal anchor domain of Nef is required for membrane association and localization into detergent-insoluble “lipid rafts” [77], while the central core encodes numerous protein interaction and intracellular trafficking motifs that contribute differentially to diverse Nef functions [78]. The central core domain of Nef adopts a stable tertiary fold, permitting its early characterization using both NMR and X-ray crystallographic methods [79,80]. Our understanding of Nef-mediated HLA down-regulation has been significantly enhanced by the recently reported crystal structure of Nef protein in complex with the MHC-I cytoplasmic domain and the μ1 subunit of the clatherin AP1 complex [76].”

The correct figure should be:
